# Supplementary material for: Impact of emergency physician-staffed ambulances on preoperative time course and survival among injured patients requiring emergency surgery or transarterial embolization: A retrospective cohort study at a community emergency department in Japan
Source: PLoS One. 2021 Nov 8;16(11):e0259733. doi: 10.1371/journal.pone.0259733 (PMC8575187; doi:10.1371/journal.pone.0259733)
Supplement: S1 Table — Data are expressed as n (%). ELST, emergency life-saving technician; EP, emergency physician; PS, propensity score. (PDF) [file pone.0259733.s002.pdf]

S1 Table. Comparison of prehospital intervention: EP-staffed ambulance versus ELST-staffed ambulance.

Prehospital intervention	ELST (n = 667)	EP (n = 353)
Endotracheal intubation	0 (0)	40 (11.3)
Cricothyrotomy	0 (0)	2 (0.6)
Tube thoracotomy or thoracentesis	0 (0)	14 (4.0)
Pericardiocentesis	0 (0)	0 (0)
Resuscitative endovascular balloon occlusion of the aorta	0 (0)	0 (0)

Data are expressed as n (%). ELST, emergency life-saving technician; EP, emergency physician; PS, propensity score.
