# Supplementary material for: Impact of emergency physician-staffed ambulances on preoperative time course and survival among injured patients requiring emergency surgery or transarterial embolization: A retrospective cohort study at a community emergency department in Japan
Source: PLoS One. 2021 Nov 8;16(11):e0259733. doi: 10.1371/journal.pone.0259733 (PMC8575187; doi:10.1371/journal.pone.0259733)
Supplement: S2 Table — Cox proportional hazards regression analyses were performed with adjustment for age, sex, Injury Severity Score, Glasgow Coma Scale score, systolic blood pressure, respiratory rate, and trauma etiology. The reference set was the group of injured patients transported to the ED by ELST-staffed ambulances. CI, confidence interval; ED, emergency department; ELST, emergency life-saving technician; EP, emergency physician; TAE, trans arterial embolization. (PDF) [file pone.0259733.s003.pdf]

S2 Table. Cox proportional hazards regression analysis of cumulative preoperative time: EP-staffed ambulance versus ELST-staffed ambulance.

Time to incidences	Hazard ratio (95% CI)	P
Time from the emergency call to ED arrival	0.29 (0.25–0.34)	<0.001
Time from ED arrival to arrival in the operating room or catheterization laboratory	1.24 (1.14–1.31)	0.048
Total time to surgery or TAE	0.78 (0.68–0.90)	0.001

Cox proportional hazards regression analyses were performed with adjustment for age, sex, Injury Severity Score, Glasgow Coma Scale score, systolic blood pressure, respiratory rate, and trauma etiology. The reference set was the group of injured patients transported to the ED by ELST-staffed ambulances.

CI, confidence interval; ED, emergency department; ELST, emergency life-saving technician; EP, emergency physician; TAE, trans arterial embolization.
