# Supplementary material for: Impact of emergency physician-staffed ambulances on preoperative time course and survival among injured patients requiring emergency surgery or transarterial embolization: A retrospective cohort study at a community emergency department in Japan
Source: PLoS One. 2021 Nov 8;16(11):e0259733. doi: 10.1371/journal.pone.0259733 (PMC8575187; doi:10.1371/journal.pone.0259733)
Supplement: S3 Table — Data are expressed as n (%) or median (interquartile range). **Adjusted standardized residual > 1.96. *Adjusted standardized residual < −1.96. aTime from the emergency call to arrival in the operating room or catheterization laboratory later than 180 min. b17:00 to 7:59 on weekdays plus all weekend hours. AIS, Abbreviated Injury Scale; ELST, emergency life-saving technician; EP, emergency physician; GCS, Glasgow Coma Scale; ISS, Injury Severity Score; NA, not available; PS, propensity score; RTS, Revised Trauma Score; SBP, systolic blood pressure; SD, standardized difference. TAE; transcatheter arterial embolization. (PDF) [file pone.0259733.s004.pdf]

S3 Table. Factors associated with delayed definitive intervention.[a]

	Time from Emergency call to surgery or TAE		P
	≤ 180 (n = 515)	> 180 (n = 505)	
Age	48.0 (26.5-64.0)	51.0 (28.0-66.0)	0.073
Gender			0.400
Male	362 (70.3)	367 (72.7)	
Female	153 (29.7)	138 (27.3)	
Trauma etiology			< 0.001
Blunt	425 (82.5)	456 (90.3)	
Penetrating	90 (17.5)	49 (9.7)	
Anatomical severity			
ISS	13.0 (9.0-27.0)	16.0 (9.0-29.0)	0.425
AIS (≥3)			
Head or neck	77 (15.0)	110 (21.8)	0.005
Face	6 (1.2)	13 (2.6)	0.096
Chest	148 (28.4)	174 (34.5)	0.049
Abdomen or pelvic contents	140 (27.2)	106 (21.0)	0.021
Extremities or pelvic girdle	276 (53.6)	282 (55.8)	0.471
Physiological parameters			
GCS score			0.063
13–15	420 (81.6)	437 (86.5)	
9–12	37 (7.2)	33 (6.5)	
6–8	25 (4.9)	21 (4.2)	
4–5	13 (2.5)	4 (0.8)	
3	20 (3.9)	10 (2.0)	
SBP, mmHg			0.001
> 89	407 (79.0)*	433 (85.7)**	
76–89	35 (6.8)	39 (7.7)	
50–75	56 (10.9)**	27 (5.3)*	
1–49	17 (3.3)**	6 (1.2)*	
Respiratory rate, breaths/min			0.057
> 29	412 (80.0)	432 (85.5)	
10–29	96 (18.6)	68 (13.5)	
6–9	4 (0.8)	4 (0.8)	

	Time from Emergency call to surgery or TAE		
	≤ 180 (n = 515)	> 180 (n = 505)	P
1–5	0 (0)	1 (0.2)	
0	3 (0.6)	0 (0)	
RTS	7.841 (6.904-7.841)	7.841 (7.550-7.841)	0.002
Probability of survival	0.980 (0.891-0.996)	0.974 (0.915-0.995)	0.563
Charlson Comorbidity Index			0.194
0	393 (76.3)	378 (74.9)	
1	89 (17.3)	82 (16.2)	
2	22 (4.3)	22 (4.4)	
≥ 3	11 (2.1)	23 (4.6)	
Presentation time			< 0.001
8:00–16:59	309 (60.0)**	265 (52.5)*	
17:00–23:59	152 (29.5)	136 (26.9)	
24:00–7:59	54 (10.5)*	104 (20.6)**	
Presentation day			0.372
Weekdays	376 (73.0)	356 (70.5)	
Weekends	139 (27.0)	149 (29.5)	
Off-hours presentation[b]	267 (51.8)*	302 (59.8)**	0.011
Type of intervention			< 0.001
TAE	133 (25.8)**	81 (16.0)*	
External skeletal fixation or open reduction with internal fixation	259 (50.3)*	343 (67.9)**	
Laparotomy or thoracotomy	100 (19.4)**	64 (12.7)*	
Craniotomy	23 (4.5)	17 (3.4)	
Dispatch of an EP-staffed ambulance	158 (30.7)	195 (38.6)	0.008

Data are expressed as n (%) or median (interquartile range).

**Adjusted standardized residual > 1.96. *Adjusted standardized residual < −1.96.

[a]Time from the emergency call to arrival in the operating room or catheterization laboratory later than 180 min.

[b]17:00 to 7:59 on weekdays plus all weekend hours.

AIS, Abbreviated Injury Scale; ELST, emergency life-saving technician; EP, emergency

physician; GCS, Glasgow Coma Scale; ISS, Injury Severity Score; NA, not available; PS, propensity score; RTS, Revised Trauma Score; SBP, systolic blood pressure; SD, standardized difference. TAE; transcatheter arterial embolization.
