# Supplementary material for: Impact of emergency physician-staffed ambulances on preoperative time course and survival among injured patients requiring emergency surgery or transarterial embolization: A retrospective cohort study at a community emergency department in Japan
Source: PLoS One. 2021 Nov 8;16(11):e0259733. doi: 10.1371/journal.pone.0259733 (PMC8575187; doi:10.1371/journal.pone.0259733)
Supplement: S4 Table — Data are expressed as n (%). ELST, emergency life-saving technician; EP, emergency physician; PS, propensity score. (PDF) [file pone.0259733.s005.pdf]

**S4 Table. Comparison of mortality rate: EP-staffed ambulance versus ELST-staffed ambulance.**

| | Full cohort | | | PS matched cohort | | |
|---|---|---|---|---|---|---|
| | **ELST (n = 667)** | **EP (n = 353)** | **P** | **ELST (n = 295)** | **EP (n = 295)** | **P** |
| **Outcome** | | | 0.002 | | | 0.671 |
| Dead | 38 (5.7) | 39 (11.0) | | 29 (9.8) | 26 (8.8) | |
| Alive | 629 (94.3) | 314 (89.0) | | 266 (90.2) | 269 (91.2) | |

Data are expressed as n (%).

ELST, emergency life-saving technician; EP, emergency physician; PS, propensity score.
