# Supplementary material for: Impact of emergency physician-staffed ambulances on preoperative time course and survival among injured patients requiring emergency surgery or transarterial embolization: A retrospective cohort study at a community emergency department in Japan
Source: PLoS One. 2021 Nov 8;16(11):e0259733. doi: 10.1371/journal.pone.0259733 (PMC8575187; doi:10.1371/journal.pone.0259733)
Supplement: S1 Fig — Distribution of PS in the full (A) and PS-matched (B) cohorts. ELST, emergency life-saving technician; EP, emergency physician; PS, propensity score. (PDF) [file pone.0259733.s006.pdf]

S1 Fig. Distribution of PS in the full (A) and PS-matched (B) cohorts.

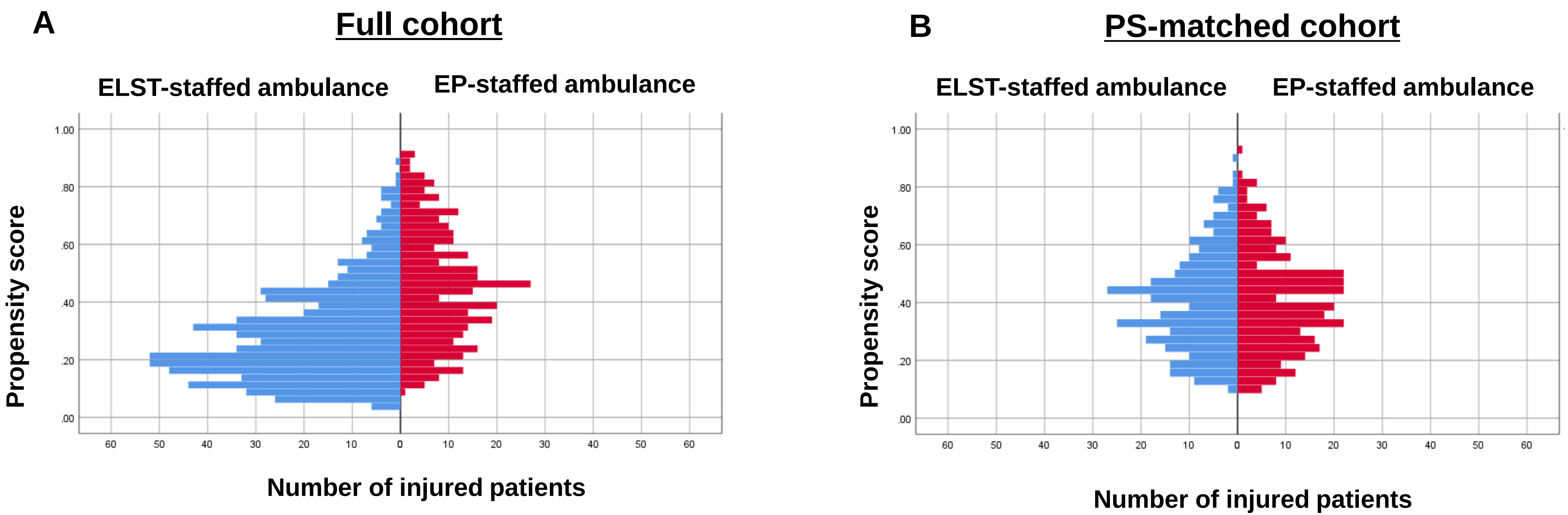

ELST, emergency life-saving technician; EP, emergency physician; PS, propensity score.
