# Supplementary material for: Impact of emergency physician-staffed ambulances on preoperative time course and survival among injured patients requiring emergency surgery or transarterial embolization: A retrospective cohort study at a community emergency department in Japan
Source: PLoS One. 2021 Nov 8;16(11):e0259733. doi: 10.1371/journal.pone.0259733 (PMC8575187; doi:10.1371/journal.pone.0259733)
Supplement: S2 Fig — Column scatter plots representing the data distribution (circles), median (horizontal bar), and interquartile range (vertical bar). **P < 0.01, *P < 0.05. The P values were derived using the Kruskal–Wallis test followed by Dunn’s post hoc tests with Bonferroni correction. EP: emergency physician; LOS: length of stay. (PDF) [file pone.0259733.s007.pdf]

S2 Fig. Prehospital LOS according to the number and type of interventions in EP-staffed ambulances.

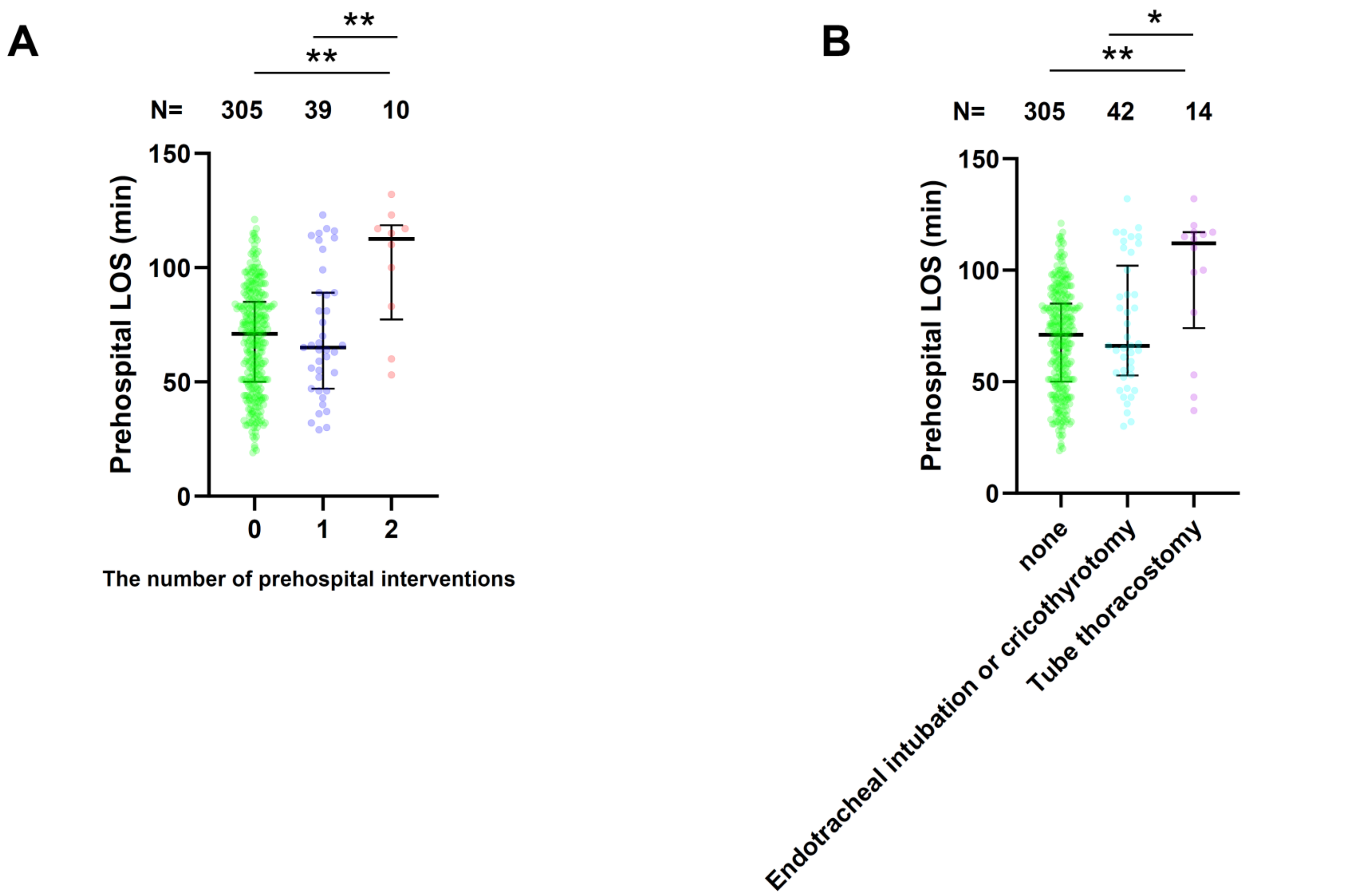

Column scatter plots representing the data distribution (circles), median (horizontal bar), and interquartile range (vertical bar).
**P < 0.01, *P < 0.05. The P values were derived using the Kruskal–Wallis test followed by Dunn's post hoc tests with Bonferroni correction.
EP: emergency physician; LOS: length of stay.
