# Supplementary material for: Impact of emergency physician-staffed ambulances on preoperative time course and survival among injured patients requiring emergency surgery or transarterial embolization: A retrospective cohort study at a community emergency department in Japan
Source: PLoS One. 2021 Nov 8;16(11):e0259733. doi: 10.1371/journal.pone.0259733 (PMC8575187; doi:10.1371/journal.pone.0259733)
Supplement: S3 Fig — Cumulative incidence rate curves from emergency call to ED arrival in the full (A) and PS-matched (B) cohorts. Cumulative incidence rate curves from ED arrival to arrival in the operating room or catheterization laboratory in the full (C) and PS-matched (D) cohorts. Cumulative incidence rate curves from emergency call to surgery or TAE in the full (E) and PS-matched (F) cohorts. ***P < 0.001, **P < 0.01, *P < 0.05 by log-rank test. ED, emergency department; ELST, emergency life-saving technician; EP, emergency physician; PS, propensity score; TAE, transarterial embolization. (PDF) [file pone.0259733.s008.pdf]

S3 Fig. Comparison of cumulative incidence rate curves: EP-staffed ambulance versus ELST-staffed ambulance.

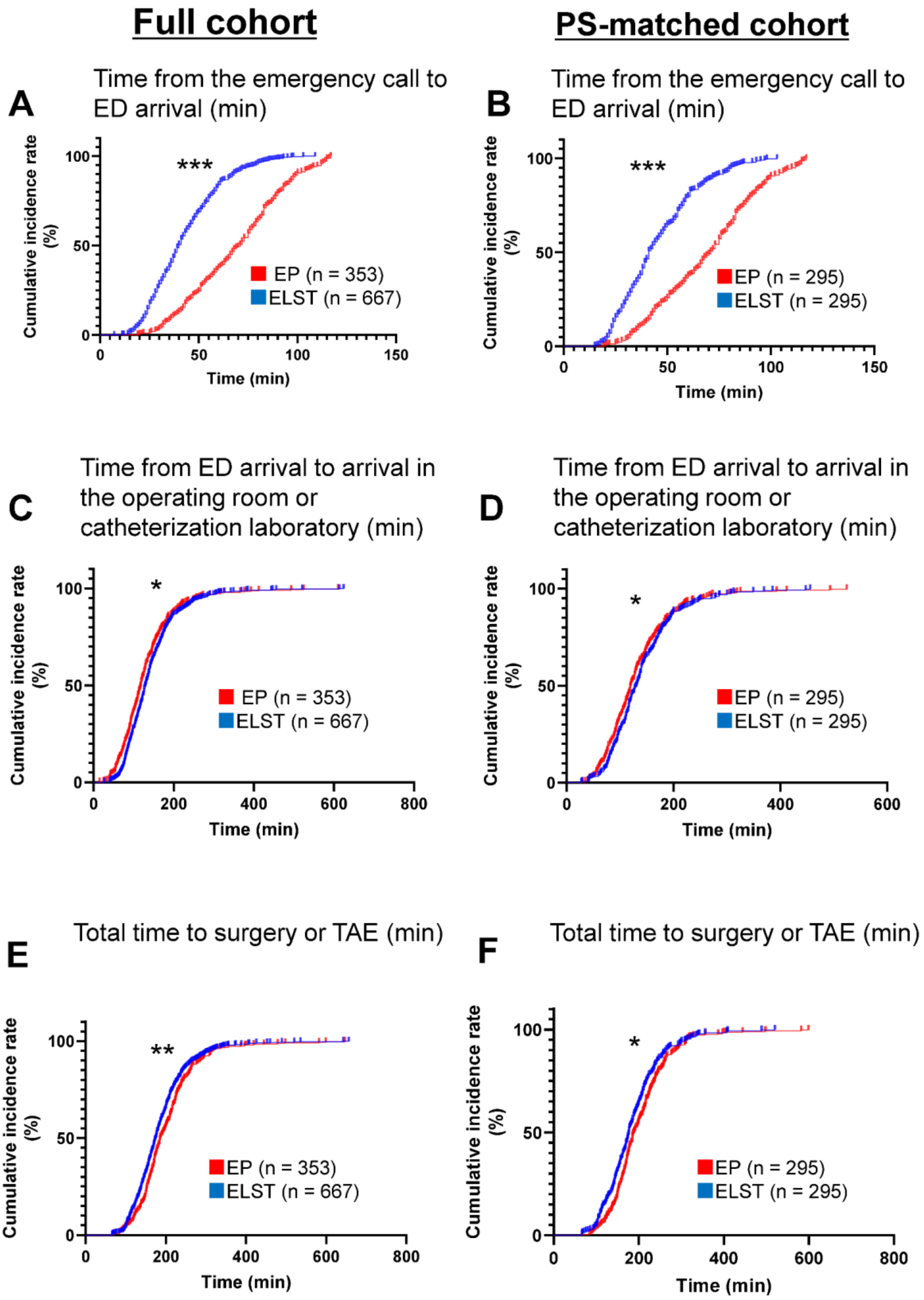

Cumulative incidence rate curves from emergency call to ED arrival in the full **(A)** and PS-matched **(B)** cohorts. Cumulative incidence rate curves from ED arrival to arrival in the operating room or catheterization laboratory in the full **(C)** and PS-matched **(D)** cohorts. Cumulative incidence rate curves from emergency call to surgery or TAE in the full **(E)** and PS-matched **(F)** cohorts.

***P < 0.001, **P < 0.01, *P < 0.05 by log-rank test.

ED, emergency department; ELST, emergency life-saving technician; EP, emergency physician; PS, propensity score; TAE, transarterial embolization.
