# Supplementary material for: Impact of emergency physician-staffed ambulances on preoperative time course and survival among injured patients requiring emergency surgery or transarterial embolization: A retrospective cohort study at a community emergency department in Japan
Source: PLoS One. 2021 Nov 8;16(11):e0259733. doi: 10.1371/journal.pone.0259733 (PMC8575187; doi:10.1371/journal.pone.0259733)
Supplement: S4 Fig — The reference set was the group of injured patients transported to the ED by ELST-staffed ambulances. aAdjusted for PS, as described in the Methods. b17:00 to 7:59 on weekdays plus all weekend hours. CI, confidence interval; ELST, emergency life-saving technician; EP, emergency physician; GCS, Glasgow Coma Scale; ISS, Injury Severity Score; OR, odds ratio; SBP, systolic blood pressure; TAE, transcatheter arterial embolization. (PDF) [file pone.0259733.s009.pdf]

S4 Fig. Subgroup analysis of hospital mortality in injured patients requiring emergency surgery or TAE: EP-staffed ambulance versus ELST-staffed ambulance.

Analysis	N	Adjusted OR (95% CI)[a]
Full cohort	1020	0.73 (0.43–1.26)
Age > 55	425	0.51 (0.26–1.01)
ISS > 15	497	0.69 (0.40–1.20)
SBP < 90mmHg	180	0.57 (0.26–1.25)
GCS score < 9	93	0.66 (0.23–1.85)
Probability of survival < 0.5	100	0.64 (0.26–1.57)
TAE	214	1.39 (0.64–3.00)
Laparotomy or thoracotomy	164	0.29 (0.10–0.85)
Off-hours presentation[b]	569	0.63 (0.30–1.33)

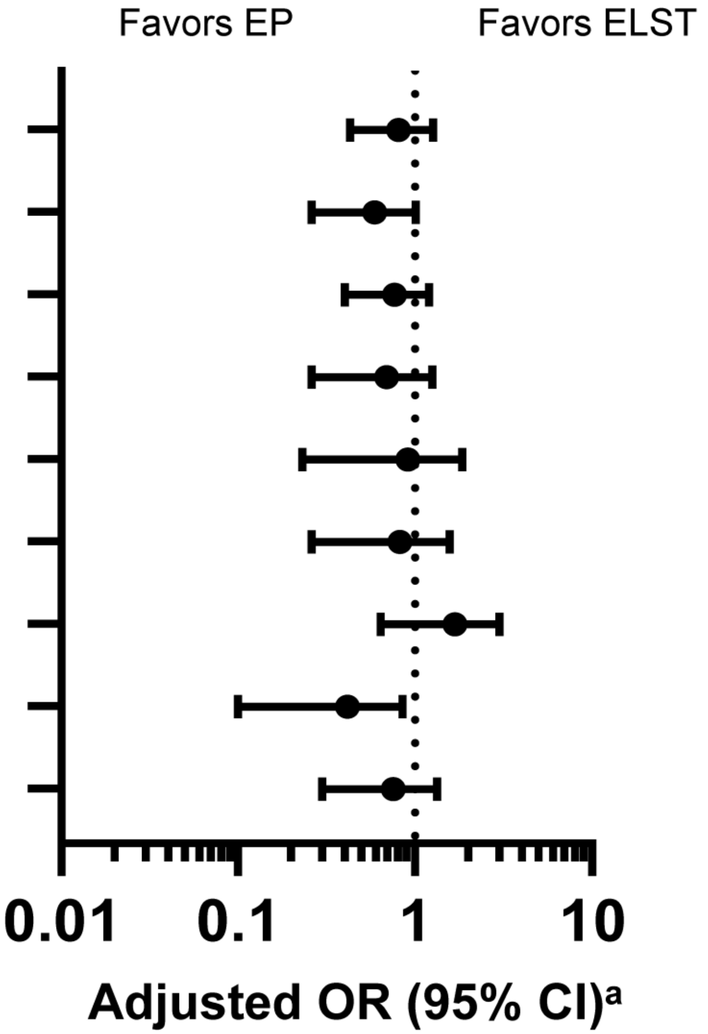

The reference set was the group of injured patients transported to the ED by ELST-staffed ambulances.
[a]Adjusted for PS, as described in the Methods.
[b]17:00 to 7:59 on weekdays plus all weekend hours.
CI, confidence interval; ELST, emergency life-saving technician; EP, emergency physician; GCS, Glasgow Coma Scale; ISS, Injury Severity Score; OR, odds ratio; SBP, systolic blood pressure; TAE, transcatheter arterial embolization.
