# Supplementary material for: Impact of emergency physician-staffed ambulances on preoperative time course and survival among injured patients requiring emergency surgery or transarterial embolization: A retrospective cohort study at a community emergency department in Japan
Source: PLoS One. 2021 Nov 8;16(11):e0259733. doi: 10.1371/journal.pone.0259733 (PMC8575187; doi:10.1371/journal.pone.0259733)
Supplement: S5 Fig — The distribution of time to death in full cohort (A) and PS-matched (B) cohort. The proportion of deaths in the full cohort (C) and PS-matched cohort (D) within 24 hours. ELST, emergency life-saving technician; EP, emergency physician; LOS, length of stay; PS, propensity score. (PDF) [file pone.0259733.s010.pdf]

S5 Fig. Time-of-death analysis: EP-staffed ambulance versus ELST-staffed ambulance.

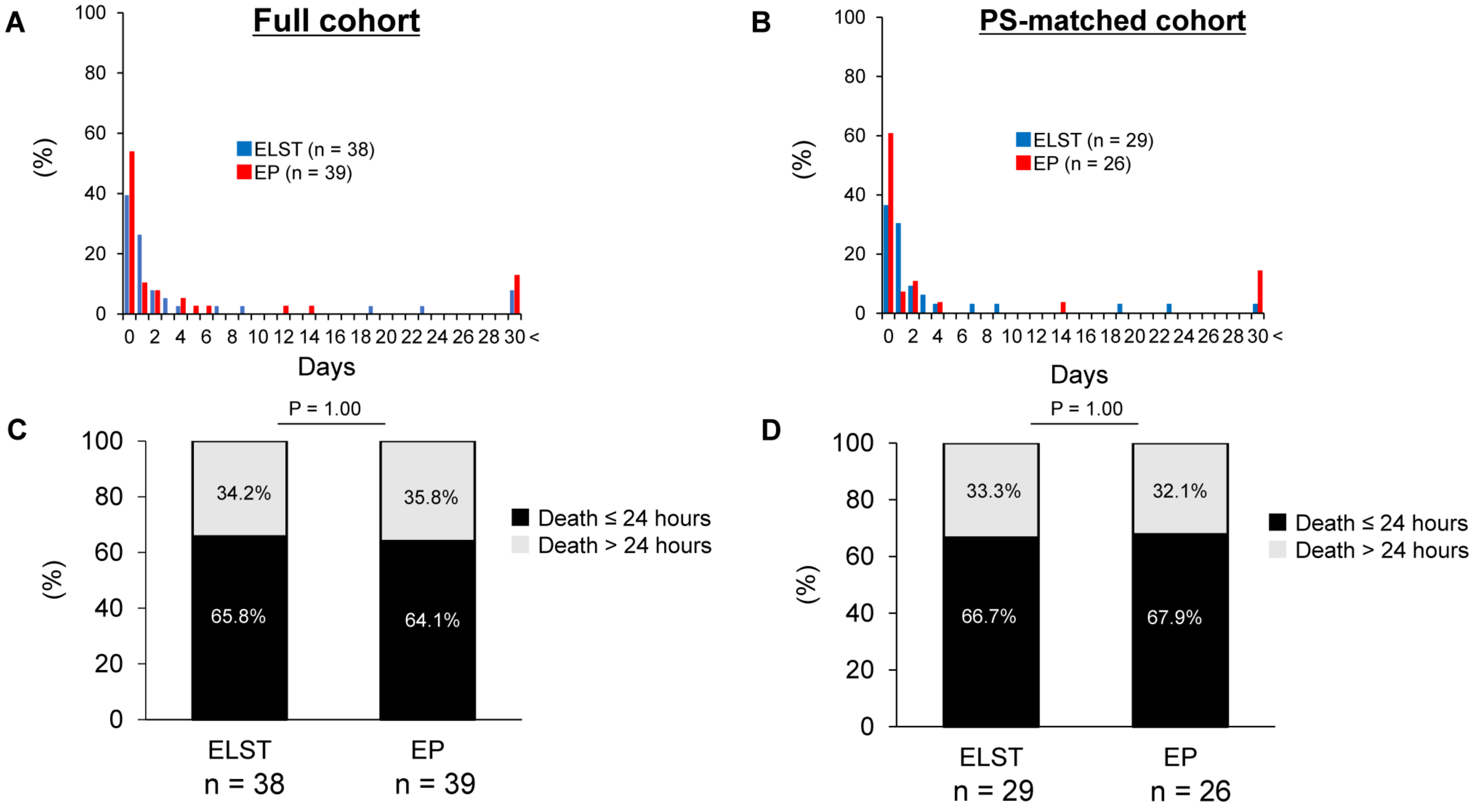

The distribution of time to death in full cohort **(A)** and PS-matched **(B)** cohort. The proportion of deaths in the full cohort (C) and PS-matched cohort (D) within 24 hours.
ELST, emergency life-saving technician; EP, emergency physician; LOS, length of stay; PS, propensity score.
